# Monitoring circulating tumor DNA liquid biopsy in stage III BRAF-mutant melanoma patients undergoing adjuvant treatment

**DOI:** 10.1186/s12967-024-05783-7

**Published:** 2024-11-28

**Authors:** Sara Marchisio, Alessia Andrea Ricci, Gabriele Roccuzzo, Eleonora Bongiovanni, Erika Ortolan, Luca Bertero, Enrico Berrino, Valentina Pala, Renata Ponti, Paolo Fava, Simona Osella-Abate, Silvia Deaglio, Caterina Marchiò, Anna Sapino, Rebecca Senetta, Ada Funaro, Simone Ribero, Pietro Quaglino, Paola Cassoni

**Affiliations:** 1 Laboratory of Immunogenetics, Department of Medical Sciences, University of Turin, Turin, Italy; 2 Pathology Unit, Department of Medical Sciences, University of Turin, Turin, Italy; 3 Section of Dermatology, Department of Medical Sciences, University of Turin, Turin, Italy; 4 Department of Medical Sciences, University of Turin, Turin, Italy; 5 Candiolo Cancer Institute, FPO-IRCCS, Candiolo, Italy; 6 Department of Oncology, Città della Salute e della Scienza of Turin, University Hospital, Turin, Italy; 7 Department of Laboratory Medicine, Città della Salute e della Scienza, University Hospital, Turin, Italy; 8 Immunogenetics and Transplant Biology Service, Città della Salute e della Scienza, Turin, Italy; 9 Pathology Unit, Department of Oncology, University of Turin, Turin, Italy

**Keywords:** Liquid biopsy, ddPCR, Cutaneous melanoma, Disease monitoring, Adjuvant therapy

## Abstract

**Background:**

The introduction of adjuvant therapies for patients with resected cutaneous melanoma (CM) has increased the need for sensitive biomarkers for risk stratification and disease monitoring. This study aims to investigate the utility of circulating tumor DNA (ctDNA) assessment in predicting and reflecting disease status during adjuvant therapy.

**Methods:**

We enrolled 32 patients with resected BRAF-mutated stage III CM receiving adjuvant targeted therapy or immunotherapy. Plasma samples of patients were collected at the baseline (treatment initiation) and during the therapy, and BRAF-mutated ctDNA was quantified by droplet digital PCR (ddPCR).

**Results:**

Baseline ctDNA was detected in 11/32 (34.4%) patients and predicted postoperative high risk of relapse [HR 3.79, 95% CI 1.20–12.00, *p* = 0.023]. The three-year overall survival (OS) rate was 54.6% (95% CI 22.9–77.9) versus 95% (95% CI 69.5–99.3) in ctDNA-positive and negative groups, respectively, with significantly worse OS for ctDNA-positive patients [HR 7.92, 95% CI 1.56–40.36, *p* = 0.013]. Among the baseline ctDNA-positive group (high-risk patients), longitudinal ctDNA detection during adjuvant therapy reflected the clinical outcomes. Only non-relapsing patients cleared their plasma ctDNA by the end of the treatment, while persistent ctDNA detection provided early evidence of disease recurrence.

**Conclusions:**

ctDNA detection shows promising results in the post-operative setting for identifying cutaneous melanoma patients at the highest risk of relapse and for real-time monitoring of patients’ clinical status and treatment response.

**Supplementary Information:**

The online version contains supplementary material available at 10.1186/s12967-024-05783-7.

## Background

Over the last decade, the incidence of melanoma has consistently risen, culminating in 324,635 new cases and 57,043 reported deaths worldwide in 2020 [1]. The introduction of innovative BRAF/MEK targeted therapies and immunotherapies in unresectable and metastatic stages has significantly reshaped the prognostic landscape for these patients [2, 3]. Additionally, their incorporation in the adjuvant setting has substantially reduced the risk of recurrence in disease-free patients at stages III and IV, as evidenced in the phase III randomized clinical trials Keynote-054, Check-Mate-238, and COMBI-AD [4–7]. Despite these remarkable therapeutic advancements, there is an ongoing need for dependable prognostic biomarkers that offer precise insights into disease progression, treatment response, and overall patient outcomes. Traditionally, prognostic biomarkers, unlike predictive ones, provide insights into overall cancer outcomes irrespective of therapy [8]. Various biomarkers, including circulating tumor cells, cell-free nucleic acids, and tumor-derived proteins, have been investigated in both preclinical and clinical settings [2, 9, 10]. Among these, circulating tumor DNA (ctDNA) stands out as the tumor-derived fraction of cell-free DNA (cfDNA), retaining the genetic and epigenetic alterations of the parental cancer cells [11]. In this scenario, its detection may provide a reliable noninvasive means of monitoring disease progression, capturing the temporal and spatial heterogeneity of the tumor through serial blood sample collection [12]. In the context of unresectable metastatic melanoma, the presence of ctDNA appears to be correlated with poor disease outcomes and diminished overall survival during systemic therapy [13, 14]. Similarly, few studies also demonstrated the utility of post-operative ctDNA detection to identify patients at high risk of relapse in resected stage III disease [15, 16]. However, most of these studies were performed before the widespread use of adjuvant BRAF/MEK inhibitors and immunotherapies. As a result, a limited number of exploratory analyses investigated the role of ctDNA in the adjuvant setting, largely restricted to adjuvant immunotherapy clinical trials [17–19]. Long et al. [19] have recently described a poorer relapse-free survival (RFS) and distant metastasis-free survival (DMFS) in stage IIIB-IV NED (no evidence of disease) patients with positive baseline ctDNA, also underlining that the combination between baseline ctDNA and tumor mutational burden or IFN-γ levels was more predictive of recurrence than any other biomarker alone. Beyond pre-treatment ctDNA, Syeda et al. [18] also demonstrated the negative prognostic impact of on-treatment ctDNA detection on disease recurrence during adjuvant immunotherapy, regardless of pre-treatment ctDNA status. In addition, the recent phase 2b clinical trial KEYNOTE-942 suggested the role of detectable baseline ctDNA as a measure of post-resection minimal residual disease in stage IIIB-IV NED patients, thus identifying those with higher relapse risk, regardless of immunotherapeutic treatment [17].

Despite the promise of ctDNA as a biomarker, its detection and quantification pose technical challenges, as ctDNA represents a small fraction (< 5% to < 0.1%) of cfDNA released by non-malignant cells [20]. Advanced techniques such as Droplet Digital PCR (ddPCR) have been developed to overcome these challenges. The simplified workflow of ddPCR offers a rapid, robust, cheap, and highly sensitive approach to detect mutations of clinical importance in cfDNA with a mutant allele frequency as low as 0.01% [12].

Here, we conducted a sensitive ddPCR analysis to detect and quantify ctDNA in plasma samples prospectively and serially collected from resected *BRAF*-mutated cutaneous melanoma (CM) patients during targeted therapy with BRAF and MEK inhibitors, or immunotherapy, in adjuvant setting. The primary goal was to investigate ctDNA prognostic implications for survival outcomes, and whether changes in ctDNA levels during adjuvant therapy can actually monitor and predict tumor response in a cohort of high-risk CM patients.

## Methods

### Patient cohort and study design

Thirty-two patients diagnosed with stage IIIA-D (according to the AJCC 8th edition guidelines) [21, 22] *BRAF*-mutated CM and treated with adjuvant therapy between 2019 and 2021 at the Pathology Unit of AOU Città della Salute e della Scienza University Hospital of Turin, Italy, were prospectively enrolled in this study. Before initiating adjuvant therapy, computed tomography (CT) scan confirmed no evidence of distant metastasis for all enrolled patients. The choice of the adjuvant regimen (i.e., BRAF-MEK inhibitors dabrafenib-trametinib or anti-PD1 nivolumab) was made through a multidisciplinary approach, aligning with the 2020 Italian Association of Medical Oncology (AIOM) guidelines [23], considering patients’ comorbidities and preferences. Adjuvant therapy was administered for one year, following current guidelines, unless there was evidence of disease progression or unacceptable toxicity. BRAF mutation analysis was previously assessed as part of the routine molecular diagnostics at the Pathology Unit. Clinical, histological, and serological data of the patients were recorded in the hospital’s database and subsequently archived in an internal computerized system based on WinSAP 3.0 software (Engineering, Rome, Italy) and TrakCare software (InterSystems Corporation: One Memorial Drive, Cambridge, MA, USA). Hematoxylin and eosin (H&E) slides from each case were reviewed by a pathologist confirming the histological type and grade by morphological characteristics. Inclusion criteria comprised individuals aged 18 years or older, with a histologically confirmed diagnosis of melanoma, and tumor stages categorized as fully resected stage IIIA-D based on AJCC 8th edition guidelines [22]. Lymph node dissection was performed in case of lymph node macroscopic involvement or in selected cases of microscopic involvement after discussion in multidisciplinary tumor board. Clinicopathological data including age at diagnosis, sex, lesion site, involved lymph nodes, metastasis, adjuvant treatment, mutational profile and follow-up data were collected from patients’ clinical reports in a dedicated and pseudonymized database based on REDCap (Research Electronic Data Capture, Vanderbilt).

### Sample collection and cfDNA extraction

Blood samples were retrieved at the beginning of adjuvant therapy (baseline) and during monthly follow-up until the end of therapy or relapse. Blood was collected in EDTA tubes and plasma was separated by centrifugation (2000x g, 15 min, 4 °C), then stored at -80 °C until extraction. All plasma samples were processed and stored in the TESEO Biobank of the Department of Medical Sciences, University of Turin (https://www.progettoeccellenzateseo.unito.it/it). cfDNA was extracted from plasma using the QIAamp MinElute ccfDNA Mini Kit (Qiagen, Hilden, Germany) according to manufacturer’s instructions, quantified using the Qubit dsDNA HS Assay kit (Invitrogen, Waltham, MA, USA) on Qubit 3.0 Fluorometer (Invitrogen) and then stored at -20 °C until processed.

### Droplet digital PCR (ddPCR)

ctDNA was quantified by QX200 Droplet Digital PCR (ddPCR) system (Bio-Rad, Hercules, CA, USA), targeting the specific BRAF mutations V600E/K/R with the commercial ddPCR BRAF V600 Screening Kit (Bio-Rad), which provides sensitive and precise detection down to 0.5% in a single well. The ddPCR reaction mixture included 10 µl of 2x ddPCR Supermix for Probes (No dUTP), 1 µl of 20x BRAF V600 Screening Assay (which includes HEX-labelled wild-type probe and FAM-labelled mutant probes), and up to 11 µl of cfDNA diluted in water according to the sample concentration, for a final volume of 22 µl. Seventy µl of the Droplet Generation Oil for Probes (Bio-Rad) was added to the ddPCR reaction mix and submitted to the QX200 Droplet Generator (Bio-Rad). PCR reaction was performed in T100 Thermal Cycler (Bio-Rad) set at the following conditions: 95 °C, 10 min; 40 cycles (94 °C, 30 s; 55 °C, 1 min); 98 °C, 10 min (all at a ramp rate of 2 °C/sec) and a final hold at 4 °C (ramp rate 1 °C/sec). Droplets were read in the QX200 Droplet Reader (Bio-Rad). Each run included genomic DNA (gDNA) extracted from healthy volunteers as wild-type control, a synthetic DNA gBlock Gene Fragment (Integrated DNA Technologies, Coralville, IA, USA) for each mutation (V600E/K/R) diluted in wild-type gDNA as positive controls and a non-template negative control. Data analysis was performed using QX Manager Standard Edition software v.1.2 (Bio-Rad). For quantification, a minimum of 10,000 acceptable droplets was required per reaction, and the threshold was set manually based on signals from positive and negative controls. Samples were classified as ctDNA positive if there was ≥ 1 positive mutant DNA droplet. The number of mutated DNA copies per reaction was used to calculate copies per milliliter of plasma (ctDNA copies/ml) using the following equation: ctDNA copies/ml = C*EV/TV/PV. C = copies/reaction (data derived from QX Manager software), EV = volume in which cfDNA was eluted (µl), TV = volume of cfDNA added to the PCR reaction (µl), PV = volume of plasma used for cfDNA extraction (ml) [24].

### Statistical analysis

RFS was defined as the duration from the initiation of adjuvant therapy to the occurrence of the first recurrence or death from any cause. OS was measured from the beginning of the adjuvant therapy until death. Data for patients who remained alive without disease recurrence or metastasis development were censored at the date of their last visit with the medical team. Associations between plasma baseline ctDNA detection and clinicopathological features were performed using Mann-Whitney, Fisher’s, and Chi-squared tests for continuous and paired nominal data, respectively. P values < 0.05 were considered statistically significant. Survival curves were generated based on the Kaplan-Meier method and analyzed through the Log-rank test. Cox proportional hazards regression models were used to explore the effects of clinical and histological variables simultaneously, according to standard statistical fitting procedures [25]. Spearman’s rank correlation coefficient was used to explore potential correlations between stage and baseline ctDNA status. Statistical analyses were performed using Stata/SE.v.18 (StataCorp, College Station, TX) and GraphPad Prism v.9.0 (GraphPad Software, CA, USA).

## Results

### Patients’ characteristics

Out of 32 patients, 15 (46.9%) were males and 17 (53.1%) were females, with a median age at diagnosis of 50 years (range: 32–80). All patients were diagnosed with stage III CM, mostly affecting the trunk (59.4%), limbs (34.4%), and head/neck (6.2%). Lymph node involvement was observed in 29/32 patients (90.6%). Basal lactate dehydrogenase (LDH) levels (normal range 250–450 UI/L) ranged from 231 to 543 UI/L in the cohort, with a median LDH level of 346 UI/L. Two (6.3%) patients exhibited high levels of LDH (> 500 UI/L). At histological revision, superficial spreading melanoma was the most common histotype (71.9%), and an epithelioid cytotype characterized all patients. The median Breslow depth was 3 mm (range: 0.5–11) and 50% of patients presented ulcerated CM, while 11/32 showed a residual nevus. Vertical growth was observed in 25 (78.1%) cases. The main clinicopathological features are resumed in Table S1 and Table S2.

All patients included in this study harbored a driving mutation of *BRAF* gene: 28/32 (87.5%) presented the p.V600E mutation, 3/32 (9.4%) the p.V600K mutation, and 1 (3.1%) patient the p.V600R mutation, respectively. Patients underwent an adjuvant treatment after surgical resection with targeted therapy against BRAF mutations (dabrafenib) plus MEK inhibitor (trametinib), except for 3/32 (9.4%) patients that were treated with anti-PD1 immunotherapy (nivolumab). The median time of adjuvant treatment was 12 months (range: 5.9–13.2). Twenty-six (81.3%) patients completed the adjuvant treatment, while 4 (12.5%) discontinued therapy due to disease progression and 2 (6.2%) because of toxicity. The median relapse-free survival (RFS) time was 36 months (range: 3–46). In our study, 8 (25%) patients died for any causes and the median overall survival (OS) was 38 months (range: 6–50).

### Prognostic significance of baseline ctDNA detection

At 36 months, 12/32 (37,5%) patients of our cohort had relapsed, and 4 of them (30.8%) within the 12-month adjuvant treatment period. In post-operative plasma samples collected before the beginning of therapy (baseline) ctDNA was detected in 11/32 patients (34.4%), with a median level of 3.2 copies/ml of plasma (range: 1.3–7.2) (Table 1).

**Table 1:** Follow-up and baseline ctDNA status of the patients (*n* = 32)

Patient ID	Clinical relapse	Time to relapse (months)	Site(s) of relapse	Death	Time to death or last follow-up (months)	Baseline ctDNA detection	Baseline ctDNA copies/ml
3	Yes	30	Lung	No	50	No	0.0
4	No	-		No	46	No	0.0
5	Yes	32	LN	Yes	41	Yes	4.2
6	Yes	23	LN, skin	No	46	Yes	1.3
13	No	-		No	45	No	0.0
14	No	-		No	44	Yes	2.0
16	No	-		No	41	No	0.0
17	No	-		Yes	45	No	0.0
18	No	-		No	46	No	0.0
20	Yes	19	LN	No	37	No	0.0
21	Yes	19	Skin	Yes	35	No	0.0
22	No	-		No	37	No	0.0
23	No	-		No	36	No	0.0
24	No	-		No	12^2^	No	0.0
32	Yes	13	Brain	Yes	13	Yes	3.2
36	No	-		No	43	Yes	6.3
37	No	-		No	37	No	0.0
40	No	-		No	38	No	0.0
41	No	-		No	41	Yes	7.2
42	Yes	5	LN, lung, liver	Yes	6	Yes	2.7
47	Yes	9	Brain	Yes	15	Yes	6.8
49	No	-		No	35	No	0.0
56	Yes	22	Widespread disease^1^	No	48	No	0.0
59	Yes	3	Brain	Yes	24	Yes	1.8
61	Yes	20	LN	No	42	No	0.0
69	No	-		No	37	No	0.0
74	No	-		No	40	No	0.0
75	No	-		No	37	No	0.0
77	No	-		No	43	Yes	2.6
80	No	-		No	35	No	0.0
82	No	-		No	35	No	0.0
85	Yes	9	Brain	Yes	10	Yes	3.3

Abbreviations: LN, lymph node; ctDNA, circulating tumor DNA

^1^patient with metastases in multiple sites: LN, lung, skin, liver, spleen, and adrenal gland. ^2^ The patient was lost at the follow-up

Clinicopathological features were similar between patients with detectable and non-detectable baseline ctDNA (Table S1). We first investigated the potential utility of baseline ctDNA detection to identify patients at high risk of relapse after surgery. At 36 months, 7 out of 11 (63.6%) patients with detectable ctDNA relapsed, compared to just 5 out of 21 (23.8%) of the undetectable ctDNA counterpart. This data suggested that basal ctDNA detection shows a trend toward predicting relapse (*p* = 0.053). To mention, all the patients who relapsed during the adjuvant therapy had detectable ctDNA at baseline. Conversely, 4/11 (36.4%) patients with detectable baseline ctDNA were still alive and recurrence-free at 36 months, compared to 12/21 (57.1%) undetectable ctDNA patients. Four out of 21 patients with negative ctDNA were censored before the end of 36-months follow-up.

**Fig. 1:**
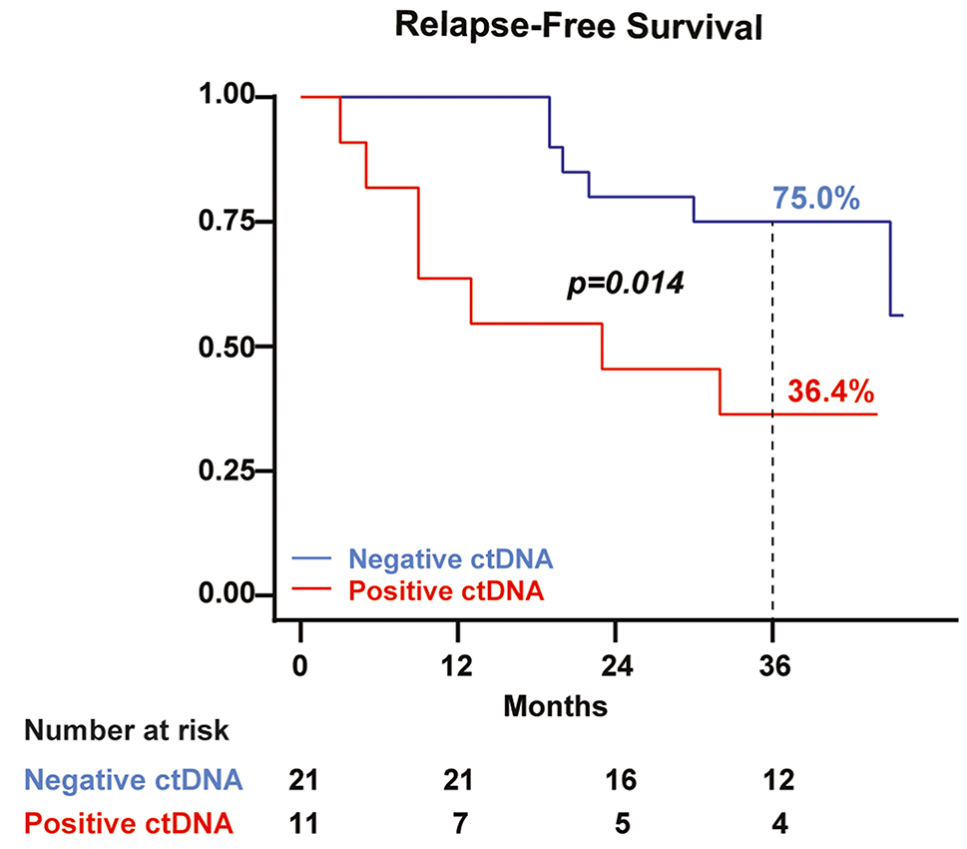
Relapse-Free Survival according to baseline ctDNA status. Kaplan-Meier analysis for relapse-free survival (RFS) in 32 CM patients stratified by baseline ctDNA detection

**Fig. 2:**
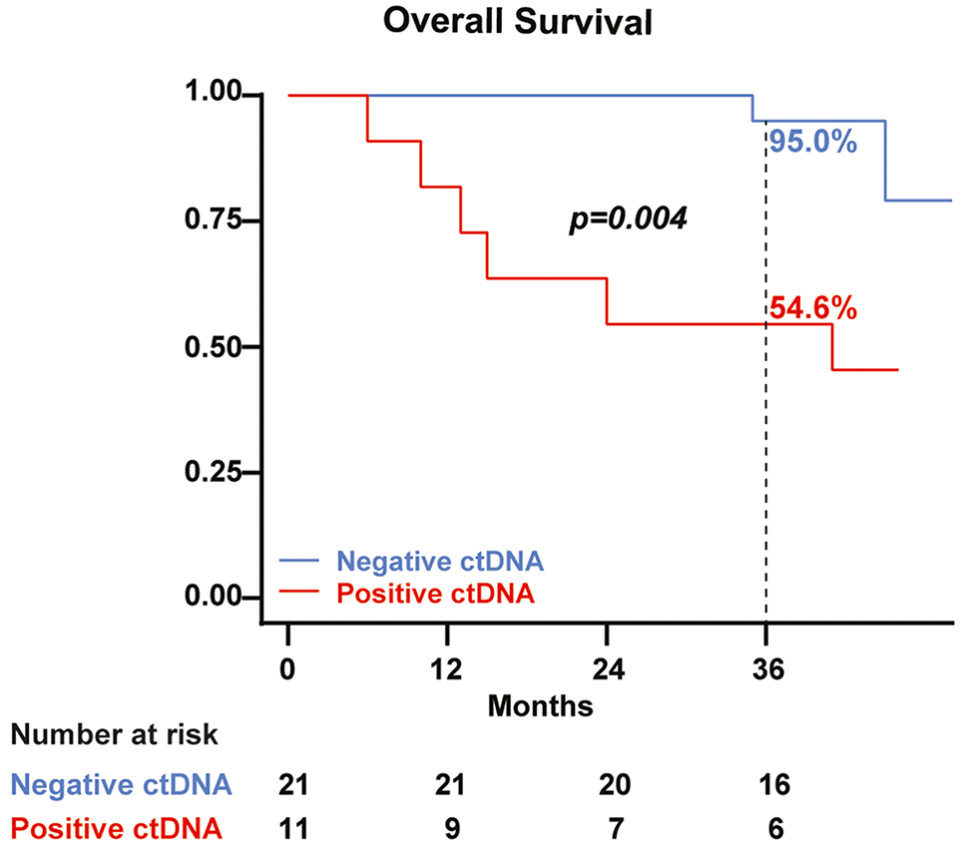
Overall Survival according to baseline ctDNA status. Kaplan-Meier analysis for overall survival (OS) in 32 CM patients stratified by baseline ctDNA detection

The 36-month RFS for the overall cohort was 61.6% (95% CI 42.3–76.1), but negative baseline ctDNA patients exhibited a significantly higher 36-month RFS of 75.0% (95% CI 50.0-88.8) compared to 36.4% (95% CI 11.2–62.7) of the positive group (*p* = 0.014) (Fig. 1). Cox univariate analysis for RFS identified in transit metastasis (HR 4.20, 95% CI 1.11–15.87, *p* = 0.034) and baseline ctDNA-positive status (HR 3.79, 95% CI 1.20–12.00, *p* = 0.023) as significant risk factors. The Log-rank test for stages didn’t show a statistically significant difference in RFS (*p* = 0.061). Next, we evaluated the predictive value of basal ctDNA on survival. The 36-month overall survival rate for the whole cohort was 80.8% (95% CI 62.2–90.9), with a significant difference observed between the positive (54.6%, 95% CI 22.9–77.9) and the negative (95.0%, 95% CI 69.5–99.3) baseline ctDNA group (*p* = 0.004) (Fig. 2). Cox univariate analysis for OS identified age (HR 1.07, 95% CI 1.01–1.14, *p* = 0.015), stage IIID (HR 8.81, 95% CI 1.68–46.21, *p* = 0.010), in transit metastasis (HR 9.44, 95% CI 1.89–47.16, *p* = 0.006), relapse during adjuvant therapy (HR 24.58, 95% CI 2.65-228.08, *p* = 0.005), brain relapse (HR 100.69, 95% CI 1.14–100.80, *p* = 0.039), and baseline ctDNA detection (HR 7.92, 95% CI 1.56–40.36, *p* = 0.013) as significant variables. The Log-rank test for stages showed statistical significance in terms of OS (*p* = 0.016). The Spearman test explored the potential correlations between stage and baseline ctDNA status. The results indicated that the baseline stage was not correlated with baseline ctDNA (*p* = 0.324). Similarly, the logistic regression, with the dependent variable being “baseline ctDNA status” and the independent variable “stage”, did not reveal any significant association (*p* = 0.1621, Pseudo R2 = 0.0993). At last, the examination of basal LDH levels demonstrated no association with relapse, death, or baseline ctDNA.

### Monitoring ctDNA dynamics for disease surveillance during adjuvant therapy

**Fig. 3:**
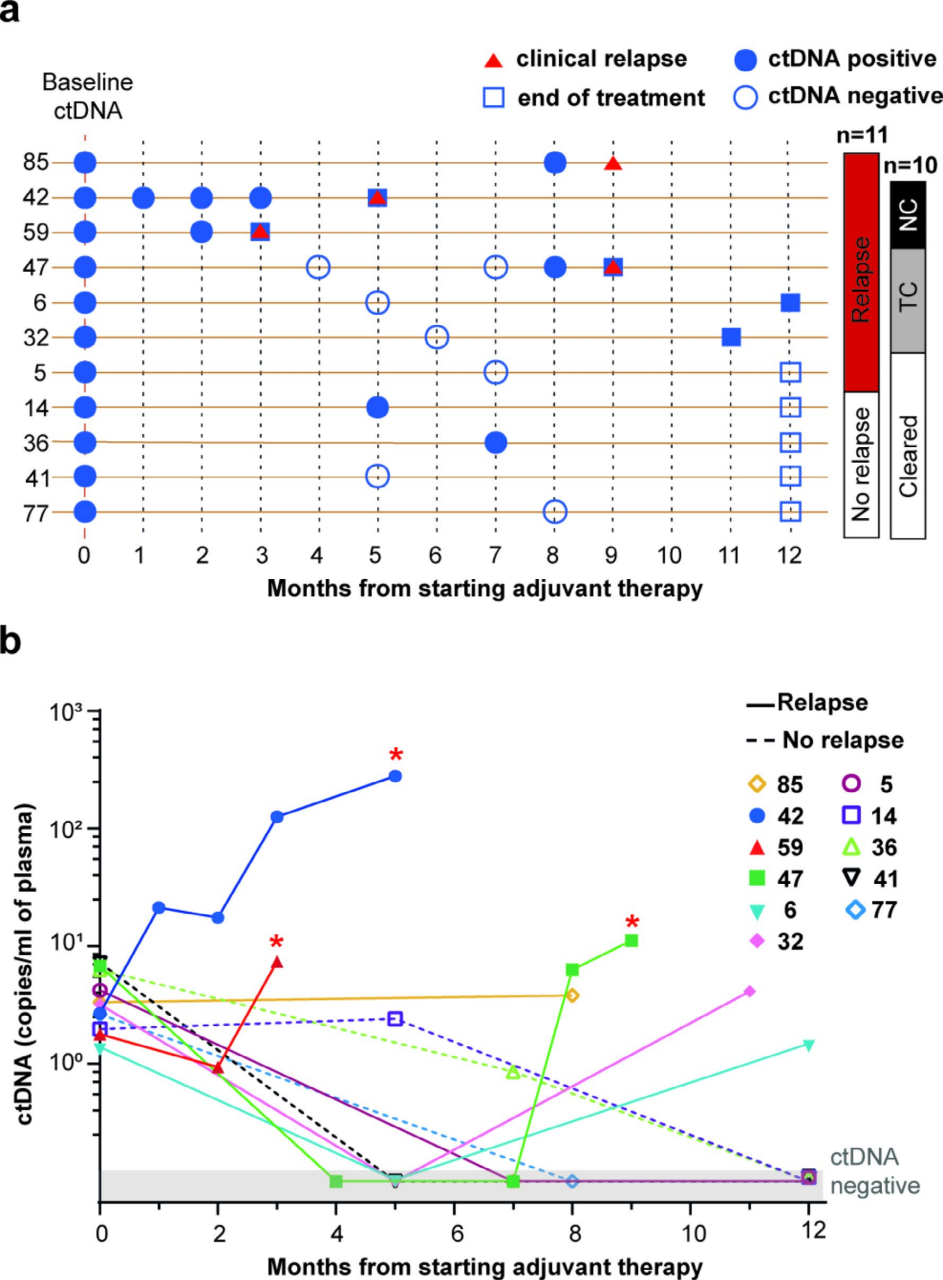
Plasma ctDNA monitoring during adjuvant therapy. **a**. Overview of plasma ctDNA assessment during adjuvant therapy (12 months) in CM patients with detectable baseline ctDNA (*n* = 11). Patients are grouped according to relapse and ctDNA clearance status. Squares indicate the plasma samples corresponding to the end of treatment, and red triangles indicate clinical relapse confirmed by imaging techniques. Empty and blue dots indicate absence and presence of ctDNA, respectively. Abbreviations: NC, non-cleared; TC, transiently cleared. **b.** Graphical representation of plasma ctDNA levels during adjuvant therapy. Each dot represents a plasma sample analyzed at the indicated time point. Dotted and plain lines depict patients who were disease-free or showed clinical relapse by the time of the analysis, respectively. * indicates the time-point overlapping with clinical evidence of disease relapse

To explore the clinical utility of ctDNA dynamics assessment during adjuvant therapy, we monitored the 11 patients with positive baseline ctDNA. During adjuvant therapy, 4 patients relapsed (Fig. 1), and all of them were previously included in the high-risk group by baseline ctDNA analysis. However, one (# 85) of these was excluded from the longitudinal analysis because the plasma sample at the time of relapse was not available. Besides the baseline, plasma was analyzed at the time of the intermediate imaging scan with CT or magnetic resonance imaging (MRI), and at the end of the therapy, which in 3/10 patients corresponded to clinical relapse (Fig. 3a). Five out of 10 patients had a transient clearance (*n* = 3) or did not clear (*n* = 2) their plasma ctDNA until adjuvant therapy ended. Noteworthy, all these patients experienced disease relapse within 36 months (Table 1). Moreover, the 3 patients who relapsed during adjuvant therapy showed higher ctDNA concentrations at relapse time compared to the 2 patients who relapsed after the end of the treatment (Fig. 3b). Conversely, the remaining 5 subjects presented a gradual decrease of plasma ctDNA levels starting from the baseline, reaching a complete clearance until treatment ended. Among these cleared patients, 4 remained disease-free until the last follow-up visit, and one (# 5) experienced loco-regional disease relapse after 32 months (Table 1).

**Fig. 4:**
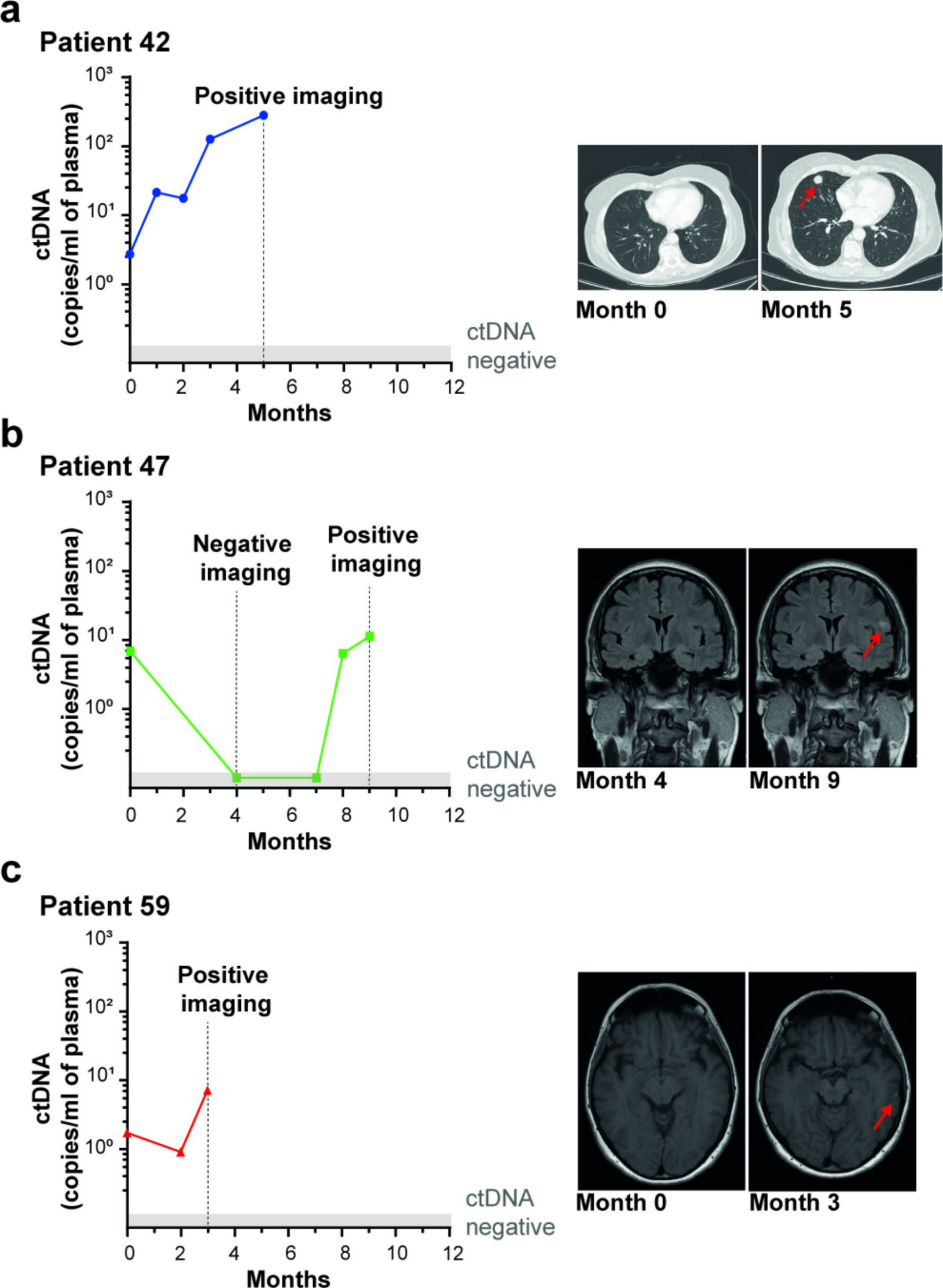
Sequential ctDNA monitoring in patients showing tumor relapse during adjuvant therapy. Graphical representation of plasma ctDNA levels in three CM patients (**a**, **b** and **c**) with evidence of disease recurrence during adjuvant therapy. Each dot represents a plasma sample analyzed. At the indicated time-point the corresponding imaging outcomes are shown. Red arrows point to metastasis

Finally, we explored the utility of serial ctDNA monitoring as an early indicator of relapse. Plasma samples of the 3 patients who relapsed during adjuvant therapy were analyzed in the months before the clinical evidence of disease recurrence (Fig. 3). The results confirmed that ctDNA was detectable before clinical recurrence. Of note, in one patient (# 42) ctDNA was persistently detectable at all time-points and its levels gradually increased until CT scan highlighted the existence of multiple extracranial lesions, including lungs (Fig. 4a), lymph nodes, and liver. In the other 2 patients (# 47 and # 59), in which the disease progression was limited to the brain, ctDNA was still able to anticipate the disease, albeit at low plasma concentrations (Fig. 4b-c).

## Discussion

In this study, we provide new insights into the prognostic value of ctDNA in resected stage III melanoma patients and emphasize the importance of serial ctDNA measurements for real-time surveillance of disease recurrence during adjuvant therapy, including BRAF/MEK targeted inhibitors.

After primary tumor eradication, ctDNA was detected in a small fraction of patients before adjuvant therapy administration; although in a small cohort, we observed that 63.6% of the ctDNA-positive patients relapsed within 36 months, and most of them experienced disease recurrence already during the year of adjuvant therapy. On the contrary, only 23.8% of ctDNA-negative patients relapsed, but none of them before the end of the treatment. While we and others showed no association between post-operative serum LDH levels and relapse or death [26], post-operative ctDNA detection was associated with an increased risk of relapse and poorer overall survival. These results suggest that post-operative ctDNA detection is a promising predictive biomarker of disease outcome, possibly indicating occult residual micrometastatic disease, which contributes to the risk of recurrence [27]. In addition, risk stratification through ctDNA assessment in resected stage III CM may distinguish between low-risk patients (ctDNA negative), who are likely to be cured by surgery alone, and high-risk patients, who may benefit from adjuvant therapy, regardless of the clinical stage and tumor. This approach can help avoid unnecessary treatment, thereby reducing both associated toxicities and costs, and can help identifying those patients who required a high-intensity surveillance.

In our high-risk group, 36.4% of patients did not relapse by the end of follow-up, possibly reflecting the effectiveness of adjuvant therapy. Assessment of ctDNA dynamics during treatment confirmed this data. Indeed, relapse-free patients showed complete clearance of plasma ctDNA by the end of the therapy, while all the subjects with permanent ctDNA detection consistently recurred. Though it is not clear why ctDNA seemed to transiently disappear in some patients, this could likely be related to the initial efficacy of adjuvant therapy on residual cancer cells [28], especially considering the longer time to first relapse in these patients compared to those with permanent ctDNA detection. These findings suggest the utility of multiple plasma ctDNA assessments to monitor treatment response and disease relapse, and prompt us to hypothesize that longer post-therapy monitoring may further increase the possibility of identifying patients with longer RFS, such as patient #5, who recurred at 32 months. In this case, ctDNA clearance might result from the long time to first relapse, but also from the locoregional nature of disease recurrence, which may not be adequately represented in plasma samples [29]. These data were supported by no baseline ctDNA detection in other patients showing only locoregional relapse (lymph nodes or skin), such as patient #61 with a lymph node progression of limited size (28 mm) at 20 months.

We serially monitored the patients who relapsed during adjuvant therapy to assess the possibility of exploiting ctDNA for early detection of recurrence. Although the analysis was performed in only 3 patients, ctDNA detection anticipated the disease in all the cases, demonstrating that ctDNA levels may faithfully reflect the course of the disease. In the patients (#47 and #59) with disease progression limited to the central nervous system, ctDNA anticipated disease recurrence, albeit at a lower plasma concentration compared to the patient with multiple lesions. Several studies have shown that the brain-blood barrier can reduce the release of ctDNA into the bloodstream, resulting in poor representation of intracranial disease by plasma ctDNA monitoring [30, 31]. The low plasma ctDNA levels in patients #47 and #59 are therefore likely due to the encephalic localization of the disease (as confirmed by imaging scans) with preservation of the brain-blood barrier integrity. Further studies in larger cohorts are needed to confirm whether ctDNA analysis can be adopted as a suitable biomarker for real-time disease surveillance, especially in patients with intracranial recurrence only.

In our cohort, the proportion of BRAF-mutated patients receiving adjuvant immunotherapy was lower than that reported in clinical trials. This prescribing trend, previously documented in Italy [32, 33], may be attributed to the fact that, following multidisciplinary evaluation, BRAF-mutated patients are typically directed towards targeted therapy.

The major limitations of the study are the low number of analyzed patients due to the single-center collection of cases, the relatively short duration of plasma collection, overlapping with the year of adjuvant treatment, and the adjuvant treatment heterogeneity. Taken together, these explain the quite low incidence of observed disease recurrences, especially considering that the expected median recurrence time in the targeted therapy adjuvant setting is associated with late-onset relapses [34]. Although the prognostic significance of ctDNA should be confirmed in a larger homogenous group of treated patients, as in the ongoing PERCIMEL study [35], we provided a pivotal pilot study focused on the monitoring of ctDNA detection before and during adjuvant therapy, including BRAF/MEK inhibitors.

## Conclusions

We demonstrated that ctDNA positivity after complete tumor eradication might help stratify CM patients according to their risk of relapse, guiding both the timing of imaging monitoring and adjuvant therapy decision-making with stricter clinical follow-ups and potentially intensified imaging protocols in high-risk patients. Furthermore, the low cost, the non-invasiveness, and the short turnaround time of ddPCR support the incorporation of ctDNA detection into the clinicopathological diagnostic workflow [15], potentially driving the decision of undergoing adjuvant therapy in challenging cases, such as in the setting of low-burden disease (e.g., stage IIIA), and for monitoring treatment response in a real-life setting.

## Electronic supplementary material

Below is the link to the electronic supplementary material.

Supplementary Material 1: Additional file 1: Table S1. Patients’ clinicopathological features according to baseline ctDNA status

Supplementary Material 2: Additional file 2: Table S2. Clinical features of patients

## Data Availability

The collected data are not publicly available to protect patients’ privacy and comply with ethical requirements. Aggregated data supporting the study findings are available from the corresponding author upon a reasonable request.

## Abbreviations

CM: Cutaneous melanoma
cfDNA: Cell-free DNA
CI: Confidence interval
ctDNA: Circulating tumor DNA
ddPCR: Droplet digital PCR
HR: Hazard ratio
LDH: Lactate dehydrogenase
OS: Overall survival
RFS: Relapse-free survival
