# Supplementary material for: Monitoring circulating tumor DNA liquid biopsy in stage III BRAF-mutant melanoma patients undergoing adjuvant treatment
Source: J Transl Med. 2024 Nov 28;22:1074. doi: 10.1186/s12967-024-05783-7 (PMC11603725; doi:10.1186/s12967-024-05783-7)
Supplement: Supplementary file 1 — Supplementary Material 1: Additional file 1: Table S1. Patients’ clinicopathological features according to baseline ctDNA status [file 12967_2024_5783_MOESM1_ESM.docx]

**SUPPLEMENTARY TABLES**

**Table S1. Patients’ clinicopathological features according to baseline ctDNA status**

|  |  | **Total (n=32)** | **ctDNA detected at baseline** | |  |
| --- | --- | --- | --- | --- | --- |
|  |  |  | **YES (n=11)** | **NO (n=21)** | **p-value^1^** |
| Sex | Male | 15 (46.9%) | 7 (63.6%) | 8 (38.1%) | 0.266 |
|  | Female | 17 (53.1%) | 4 (36.4%) | 13 (61.9%) |  |
| Age at diagnosis (years) | Median | 50 | 50 | 50 | 0.961 |
|  | Interval | 32-80 | 36-80 | 32-75 |  |
| Stage | IIIA | 3 (9.4%) | 2 (18.2%) | 1 (4.8%) | 0.05 |
|  | IIIB | 9 (28%) | 1 (9.1%) | 8 (38.1%) |  |
|  | IIIC | 18 (56.3%) | 6 (54.5%) | 12 (57.1%) |  |
|  | IIID | 2 (6.3%) | 2 (18.2%) | 0 |  |
| Tumor site | Limbs | 11 (34.4%) | 4 (36.4%) | 7 (33.3%) | ≥1 |
|  | Trunk | 19 (59.4%) | 6 (54.5%) | 13 (61.9%) |  |
|  | Head/neck | 2 (6.2%) | 1 (9.1%) | 1 (4.8%) |  |
| Metastasis in transit | Present | 4 (12.5%) | 2 (18.2%) | 2 (9.5%) | 0.593 |
|  | Absent | 28 (87.5%) | 9 (81.8%) | 19 (90.5%) |  |
| Histotype | SSM | 23 (71.9%) | 9 (81.8%) | 14 (66.6%) | 0.682 |
|  | NM | 6 (18.8%) | 1 (9.1%) | 5 (23.8%) |  |
|  | AM | 2 (6.3%) | 1 (9.1%) | 1 (4.8%) |  |
|  | Missing | 1 (3%) | 0 | 1 (4.8%) |  |
| LDH (UI/L)^2^ | Median | 346 | 375 | 336 | 0.116 |
|  | Interval | 231-543 | 293-412 | 231-543 |  |
|  | High | 2 (6.3%) | 0 | 2 (9.5%) | 0.527 |
|  | Low | 29 (90.6%) | 11 (100%) | 18 (85.7%) |  |
|  | Missing | 1 (3.1%) | 0 | 1 (4.8%) |  |
| Clark level | III | 11 (34.4%) | 4 (36.4%) | 7 (33.3%) | 0.508 |
|  | IV | 19 (59.4%) | 6 (54.5%) | 13 (61.9%) |  |
|  | V | 1 (3.1%) | 1 (9.1%) | 0 |  |
|  | Missing | 1 (3.1%) | 0 | 1 (4.8%) |  |
| Breslow depth (mm) | Median  Interval | 3  0.5-11 | 3 | 3 | 0.677 |
|  |  |  | 0.8-11 | 0.5-8 |  |
| Ulceration | Present | 16 (50%) | 6 (54.5%) | 10 (47.6%) | 0.809 |
|  | Absent | 15 (46.9%) | 5 (45.5%) | 10 (47.6%) |  |
|  | Missing | 1 (3.1%) | 0 | 1 (4.8%) |  |
| Residual nevus | Present | 11 (34.4%) | 4 (36.4%) | 7 (33.3%) | ≥1 |
|  | Absent | 18 (56.2%) | 7 (63.6%) | 11 (52.4%) |  |
|  | Missing | 3 (9.4%) | 0 | 3 (14.3%) |  |
| Vertical growth | Present | 25 (78.1%) | 9 (81.8%) | 16 (76.2%) | 0.622 |
|  | Absent | 4 (12.5%) | 2 (18.2%) | 2 (9.5%) |  |
|  | Missing | 3 (9.4%) | 0 | 3 (14.3%) |  |
| Mitosis | Median | 7 | 8 | 7 | 0.443 |
|  | Interval | 0-18 | 1-18 | 0-18 |  |
| TILs | Present (Brisk) | 2 (6.2%) | 2 (18.2%) | 0 | 0.277 |
|  | Present (not Brisk) | 18 (56.3%) | 7 (63.6%) | 11 (52.4%) |  |
|  | Poor infiltrate | 2 (6.2%) | 0 | 2 (9.5%) |  |
|  | Absent | 7 (21.9%) | 2 (18.2%) | 5 (23.8%) |  |
|  | Missing | 3 (9.4%) | 0 | 3 (14.3%) |  |
| Regression | Present (<75%) | 13 (40.6%) | 7 (63.6%) | 6 (28.6%) | 0.132 |
|  | Absent | 17 (53.1%) | 4 (36.4%) | 13 (61.9%) |  |
|  | Missing | 2 (6.3%) | 0 | 2 (9.5%) |  |
| Vascular invasion | Present | 7 (21.9%) | 1 (9.1%) | 6 (28.6%) | 0.372 |
|  | Absent | 24 (75%) | 10 (90.9%) | 14 (66.6%) |  |
|  | Missing | 1 (3.1%) | 0 | 1 (4.8%) |  |
| LN involvement | Microscopic | 25 (78.1%) | 8 (72.7%) | 17 (81%) | 0.810 |
|  | Macroscopic | 4 (12.5%) | 2 (18.2%) | 2 (9.5%) |  |
|  | Absent | 3 (9.4%) | 1 (9.1%) | 2 (9.5%) |  |
| Positive sentinel LN | Yes | 25 (78.1%) | 8 (72.7%) | 17 (81%) | 0.592 |
|  | No | 4 (12.5%) | 2 (18.2%) | 2 (9.5%) |  |
|  | Missing | 3 (9.4%) | 1 (9.1%) | 2 (9.5%) |  |
| LN Site | Axillary | 15 (46.9%) | 6 (54.5%) | 9 (42.8%) | 0.449 |
|  | Inguinal | 10 (31.3%) | 2 (18.2%) | 8 (38.1%) |  |
|  | Laterocervical | 1 (3.1%) | 0 | 0 |  |
|  | Supraclavicular | 1 (3.1%) | 1 (9.1%) | 1 (4.8%) |  |
|  | Submammary | 1 (3.1%) | 0 | 1 (4.8%) |  |
|  | Missing | 4 (12.5%) | 2 (18.2%) | 2 (9.5%) |  |
| LN dissection | Yes | 19 (59.4%) | 8 (72.7%) | 11 (52.4%) | 0.450 |
|  | No | 13 (40.6%) | 3 (27.3%) | 10 (47.6%) |  |

^1^Significant p-value: ≤0.05; ^2^Cut-off: ≥500 UI/L

Abbreviations: TILS, tumor infiltrating lymphocytes; LDH, lactate dehydrogenase; SSM, superficial spreading melanoma; NM, nodular melanoma; AM, acral melanoma; LN, lymph node; ctDNA, circulating tumor DNA.
