# Supplementary material for: Monitoring circulating tumor DNA liquid biopsy in stage III BRAF-mutant melanoma patients undergoing adjuvant treatment
Source: J Transl Med. 2024 Nov 28;22:1074. doi: 10.1186/s12967-024-05783-7 (PMC11603725; doi:10.1186/s12967-024-05783-7)
Supplement: Supplementary file 2 — Supplementary Material 2: Additional file 2: Table S2. Clinical features of patients [file 12967_2024_5783_MOESM2_ESM.docx]

**Table S2: Clinical features of patients (n=32)**

| **Patient ID** | **Age at diagnosis** | **Sex** | **Stage** | **Adjuvant treatment** |  |
| --- | --- | --- | --- | --- | --- |
| 3 | 50 | F | IIIB | TT |  |
| 4 | 32 | F | IIIC | TT |  |
| 5 | 53 | M | IIID | TT |  |
| 6 | 37 | M | IIIC | TT |  |
| 13 | 56 | F | IIIB | TT |  |
| 14 | 70 | F | IIIC | TT |  |
| 16 | 45 | F | IIIB | TT |  |
| 17 | 73 | M | IIIC | ICI |  |
| 18 | 41 | M | IIIA | TT |  |
| 20 | 58 | F | IIIC | TT |  |
| 21 | 60 | M | IIIB | TT |  |
| 22 | 57 | F | IIIC | TT |  |
| 23 | 54 | M | IIIC | TT |  |
| 24 | 63 | M | IIIB | TT |  |
| 32 | 80 | M | IIIC | TT |  |
| 36 | 44 | M | IIIA | TT |  |
| 37 | 32 | F | IIIC | TT |  |
| 40 | 75 | M | IIIC | TT |  |
| 41 | 36 | F | IIIB | TT |  |
| 42 | 78 | F | IIID | ICI |  |
| 47 | 50 | M | IIIC | TT |  |
| 49 | 60 | F | IIIC | TT |  |
| 56 | 49 | F | IIIC | TT |  |
| 59 | 43 | F | IIIC | TT |  |
| 61 | 35 | F | IIIC | TT |  |
| 69 | 49 | M | IIIC | ICI |  |
| 74 | 43 | F | IIIB | TT |  |
| 75 | 51 | M | IIIB | TT |  |
| 77 | 47 | M | IIIA | TT |  |
| 80 | 50 | F | IIIC | TT |  |
| 82 | 45 | F | IIIB | TT |  |
| 85 | 51 | M | IIIC | TT |  |

Abbreviations: TT, Targeted therapy (Dabrafenib+Trametinib), ICI, Immunotherapy (Nivolumab)
